# Integrating tropical research into biology education is urgently needed

**DOI:** 10.1371/journal.pbio.3001674

**Published:** 2022-06-16

**Authors:** Ann E. Russell, T. Mitchell Aide, Elizabeth Braker, Carissa N. Ganong, Rebecca D. Hardin, Karen D. Holl, Sara C. Hotchkiss, Jeffrey A. Klemens, Erin K. Kuprewicz, Deedra McClearn, George Middendorf, Rebecca Ostertag, Jennifer S. Powers, Sabrina E. Russo, Jennifer L. Stynoski, Ursula Valdez, Charles G. Willis

**Affiliations:** 1 Department of Natural Resource Ecology & Management, Iowa State University, Ames, Iowa, United States of America; 2 Department of Biology, University of Puerto Rico—Rio Piedras, San Juan, Puerto Rico; 3 Biology Department, Occidental College, Los Angeles, California, United States of America; 4 Organization for Tropical Studies, Durham, North Carolina, United States of America; 5 Department of Biology, Missouri Western State University, St. Joseph, Missouri, United States of America; 6 School for Environment and Sustainability, University of Michigan, Ann Arbor, Michigan, United States of America; 7 Environmental Studies Department, University of California, Santa Cruz, California, United States of America; 8 Department of Botany, University of Wisconsin, Madison, Wisconsin, United States of America; 9 Department of Biological and Chemical Sciences, Thomas Jefferson University, Philadelphia, Pennsylvania, United States of America; 10 Connecticut State Museum of Natural History, Institute of the Environment, University of Connecticut, Storrs, Connecticut, United States of America; 11 Department of Ecology & Evolutionary Biology, University of Connecticut, Storrs, Connecticut, United States of America; 12 Department of Biology, Howard University, Washington DC, United States of America; 13 Department of Biology, University of Hawaiʻi, Hilo, Hawaiʻi, United States of America; 14 Departments of Ecology, Evolution, & Behavior and Plant & Microbial Biology, University of Minnesota, Minneapolis, Minnesota, United States of America; 15 School of Biological Sciences, University of Nebraska-Lincoln, Lincoln, Nebraska, United States of America; 16 Center for Plant Science Innovation, University of Nebraska-Lincoln, Lincoln, Nebraska, United States of America; 17 Instituto Clodomiro Picado, Universidad de Costa Rica, San José, Costa Rica; 18 School of Interdisciplinary Arts & Sciences, University of Washington, Bothell, Washington, United States of America; 19 Department of Biology Teaching and Learning, University of Minnesota, Minneapolis, Minnesota, United States of America

## Abstract

Tropical biology is currently largely absent from undergraduate curricula. The integration of authentic research in tropical biology could promote internationality among scientists and provide experiential learning that enables students to address complex global problems.

## Teaser

Understanding tropical biology is important for solving complex problems such as climate change, biodiversity loss, and zoonotic pandemics, but biology curricula view research mostly via a temperate-zone lens. Integrating tropical research into biology education is urgently needed to tackle these issues.

The tropics are engines of Earth systems that regulate global cycles of carbon and water, and are thus critical for management of greenhouse gases. Compared with higher-latitude areas, tropical regions contain a greater diversity of biomes, organisms, and complexity of biological interactions. The tropics house the majority of the world’s human population and provide important global commodities from species that originated there: coffee, chocolate, palm oil, and species that yield the cancer drugs vincristine and vinblastine. Tropical regions, especially biodiversity hotspots, harbor zoonoses, thereby having an important role in emerging infectious diseases amidst the complex interactions of global environmental change and wildlife migration [1]. These well-known roles are oversimplified, but serve to highlight the global biological importance of tropical systems.

Despite the importance of tropical regions, biology curricula worldwide generally lack coverage of tropical research. Given logistical, economic, or other barriers, it is difficult for undergraduate biology instructors to provide their students with field-based experience in tropical biology research in a diverse range of settings, an issue exacerbated by the Coronavirus Disease 2019 (COVID-19) pandemic. Even in the tropics, field-based experience may be limited to home regions. When tropical biology is introduced in curricula, it is often through a temperate-zone lens that does not do justice to the distinct ecosystems, sociopolitical histories, and conservation issues that exist across tropical countries and regions [2]. The tropics are often caricatured as distant locations known for their remarkable biodiversity, complicated species interactions, and unchecked deforestation. This presentation, often originating from a colonial and culturally biased perspective, may fail to highlight the role of tropical ecosystems in global environmental and social challenges that accompany rising temperatures, worldwide biodiversity loss, zoonotic pandemics, and the environmental costs of ensuring food, water, and other ecosystem services for humans [3].

Of the 151 biology-focused Research Experience for Undergraduate (REU) grants awarded by the US National Science Foundation, only 5 (3.3%) are at least partially focused on tropical biology (correct as of May 2022). More troubling is the limited extent to which the tropics are included in textbooks, and other teaching materials in both temperate-based and tropics-based institutions. Among those of us working at institutions in tropical countries, we note anecdotally that place-based and culturally relevant educational material is often lacking, given that directly translated versions of textbooks written by temperate-based authors for temperate-based students form the basis of many undergraduate biology curricula [4]. Furthermore, in a scan of 24 websites with open-access science educational modules in 2019, we (EK, JS, JK) found that only 2% (91/4,864) of modules contained a focus on the tropics.

Life science undergraduates must currently be prepared to address complex problems without clear solutions that require higher-level thinking about connections across scales of organization, disciplines, and within both local and global contexts. When tropical ecology is presented in the context of its full biological, environmental, social, and economic complexity, students gain an excellent opportunity to study and understand such problems, such as recognizing how the impacts of colonialism are observable at political and economic levels, and within the process of science itself. We envision that students who have learned this history are more likely to support training and capacity-building of local community members and researchers, thereby promoting an inclusive, equitable, and collaborative global community [5].

How can we bridge the gaps that prevent research in tropical biology from being integrated into undergraduate biology curricula? Stahl and colleagues [6], noting the dearth of conservation biology textbooks published outside of North America and Europe, urged authors to expand their networks and seek collaborations from different geographic areas. They also called for publishers to demand examples from a greater range of regions, taxa, and scientists. This more global perspective in the primary literature could then be incorporated into textbooks. Currently, in the field of ecology, the goal is to engage in multiple practices that promote establishment of diverse, inclusive teams to empower participants in creating new knowledge and decolonize access and expertise in conducting and publishing research [7].

Another medium—online, open-educational resources (OERs)—has the potential to inspire undergraduate students, particularly if OERs are accessible, interactive, and based on authentic field research. As members of a new network for facilitating Online Content for Experiential Learning of Tropical Systems (https://ocelots.nrem.iastate.edu/), we invite readers to join a community that brings together researchers in tropical biology and experts in media, software innovation, and new pedagogy. This network includes the Ecological Society of America’s Four-Dimensional Ecological Education (4DEE) framework [8] in creating online modules. Our participatory process aims to broaden international collaboration and engage participants early on, to allow for true co-construction of knowledge. Because researchers may lack experience in creating OERs, a critical component in OCELOTS is the platform itself—Gala—intentionally designed to be user-friendly, open-source, and open-access, such that anyone with internet access can author a module [9] (examples in Box 1).

**Box 1. Examples of OCELOTS modules**

- “Healing the Scars: Tropical rainforest carbon cycling—Does it matter which tree species you plant?”
  ○ A long-term field experiment in Costa Rica connects the dots about how carbon cycling traits of species at the whole-plant level translate into global-level effects.
  ○ https://www.learngala.com/cases/tropical-rainforest-carbon-cycling
- “Restoring tropical forests”
  ○ This case study of tropical forest restoration in Costa Rica (in English and Spanish) lets students interpret bar graphs, use R Shiny to visualize seed rain in different restoration treatments, and learn about social obstacles to restoration.
  ○ https://www.learngala.com/cases/restoring-tropical-forests
  ○ https://www.learngala.com/cases/restaurando-bosques-tropicales
- “Snapshot Serengeti Online Lab”
  ○ This multi-week inquiry lets students explore African wildlife ecology by interactively analyzing camera trap data.
  ○ https://www.learngala.com/cases/snapshot-serengeti-preview-version
- “Sounds of the tropics”
  ○ This 3-part series begins with understandings of recorded wildlife sounds, then shows how such auditory data can serve ecological and conservation studies.
  ○ https://www.learngala.com/cases/sounds-of-the-tropics-part-1

We recognize that there is no substitute for in-person field experiences, but OERs provide opportunities for students who would otherwise not discover the diverse range of tropical research settings. OERs can also complement field-based experiences, allowing students to compare multiple locations and gain insights into authentic research beyond their campus. We posit that online modules created by tropical researchers themselves will bring the flavor and excitement of authentic research into the classroom in a way that sparks interest in global biological issues, without requiring travel. Online modules that can be easily created by researchers, and that immerse learners in real-world tropical research are urgently needed.

Integrating tropical biology into biology curricula will facilitate teaching within a global context that allows instructors to illustrate and emphasize the diversity and internationality of the scientific community [10]. We envision that exposure to tropical biology and local-to-global connections will engender an outward-looking, reflective perspective that promotes metacognitive skills that are fundamental for lifelong learning and development. Educational experiences that incorporate real research, data analysis, and global perspectives into biology courses worldwide will prepare students to tackle complex problems, while providing ways to develop core competencies in biology that transcend disciplines and grow foundations for excellence in diverse career paths.

## Abbreviations

COVID-19: Coronavirus Disease 2019
OCELOTS: Online Content for Experiential Learning of Tropical Systems
OER: open-educational resource
REU: Research Experience for Undergraduate
4DEE: Four-Dimensional Ecological Education
