# Correction: Integrating tropical research into biology education is urgently needed

**DOI:** 10.1371/journal.pbio.3001894

**Published:** 2022-11-11

**Authors:** Ann E. Russell, T. Mitchell Aide, Elizabeth Braker, Emilio M. Bruno, Carissa N. Ganong, Rebecca D. Hardin, Karen D. Holl, Sara C. Hotchkiss, Jeffrey A. Klemens, Erin K. Kuprewicz, Deedra McClearn, George Middendorf, Rebecca Ostertag, Jennifer S. Powers, Sabrina E. Russo, Jennifer L. Stynoski, Ursula Valdez, Charles G. Willis

Dr. Emilio M. Bruno is not included in the author byline. He should be listed as the fourth author, and his affiliation is: Center for Latin American Studies, University of Florida, Gainesville, Florida, and Department of Wildlife Ecology & Conservation, University of Florida, Gainesville, Florida, United States of America. The contributions of this author are as follows: Conceptualization, Writing–original draft.

The correct citation is: Russell AE, Aide TM, Braker E, Bruno EM, Ganong CN, Hardin RD, et al. (2022) Integrating tropical research into biology education is urgently needed. PLoS Biol 20(6): e3001674. https://doi.org/10.1371/journal.pbio.3001674
