# Correction: Correction: Integrating tropical research into biology education is urgently needed

**DOI:** 10.1371/journal.pbio.3001928

**Published:** 2022-12-02

**Authors:** The PLOS Biology Staff

There is a typographical error in the spelling of the fourth author’s name in the correction published on August 11, 2022. The correct spelling is: Emilio M. Bruna.

The correct citation of the original article is:

Russell AE, Aide TM, Braker E, Bruna EM, Ganong CN, Hardin RD, et al. (2022) Integrating tropical research into biology education is urgently needed. PLoS Biol 20(6): e3001674. https://doi.org/10.1371/journal.pbio.3001674

The publisher apologizes for the error.
